# Machine learning-based risk prediction for invasive pulmonary aspergillosis in non-neutropenic intensive care unit patients, with an exploratory analysis of pathogen-related risk factors

**DOI:** 10.3389/fcimb.2026.1967577

**Published:** 2026-10-14

**Authors:** Jijia Bai, Tao Feng, Bofei Liu, Jinyan Wang, Zhifeng Wang, Furui Liu, Jinyuan Zhu

**Affiliations:** 1 Department of Critical Care Medicine, General Hospital of Ningxia Medical University, Yinchuan, China; 2 The Third People’s Hospital of Ningxia, Yinchuan, Ningxia, China; 3 The Fourth People’s Hospital of Ningxia Hui Autonomous Region, Yinchuan, Ningxia, China; 4 The First School of Clinical Medicine, Ningxia Medical University, Yinchuan, Ningxia, China

**Keywords:** corticosteroids, gram-negative bacteria, invasive pulmonary aspergillosis, machine learning, non-neutropenic, *Pseudomonas aeruginosa*

## Abstract

**Background:**

Invasive pulmonary aspergillosis (IPA) is increasingly recognized in non-neutropenic patients, but validated bedside risk stratification tools remain scarce. We developed a machine learning model to predict IPA and tested whether corticosteroid effect depends on bacterial co-pathogen.

**Methods:**

This multicenter retrospective cohort study enrolled 837 non-neutropenic adults hospitalized with pulmonary infections across three tertiary hospitals in China between December 2020 and December 2025. The discovery cohort was split into training and internal validation sets. Four classifiers were compared, with Random Forest selected for optimal performance. The final model was externally validated in an independent cohort. Feature importance was assessed using SHAP (SHapley Additive exPlanations) analysis. Corticosteroid-bacteria interactions were examined using subgroup analyses and propensity score matching, with the synergy index S calculated to quantify biological interaction. A simplified bedside score was derived from the top-ranking predictors.

**Results:**

The Random Forest model achieved external validation AUC of 0.865. Six predictors consistently dominated across all assessments: *Pseudomonas aeruginosa* isolation, hypoxemia (PaO_2_/FiO_2_ <200 mmHg), diabetes mellitus, organ failure (Sequential Organ Failure Assessment, SOFA >5), lymphopenia (lymphocyte count <0.8×10^9^/L), and ICU (intensive care unit) admission. Corticosteroid exposure did not rank among the top six predictors. However, its association with IPA diverged sharply by bacterial context: OR below 1 with *P. aeruginosa* (OR 0.52, 95% CI 0.214–1.283) but OR above 1 with other Gram-negative bacilli (OR 2.17, 95% CI 0.722–6.535); P for interaction = 0.131; a directional pattern that persisted after propensity matching. The six-variable score derived from these predictors showed AUCs of 0.869 (training), 0.774 (internal validation), and 0.719 (external validation).

**Conclusions:**

IPA risk in non-neutropenic ICU patients is shaped more by bacterial co-pathogen context than by corticosteroid exposure alone. The divergent corticosteroid effects across bacterial species challenge the conventional assumption of a uniform steroid-associated risk. The externally validated six-variable score offers a practical, software-free tool to support pathogen-informed, individualized risk stratification and clinical decision-making in the ICU.

## Introduction

The lung represents the primary portal of entry for *Aspergillus fumigatus*, a ubiquitous environmental mold that causes invasive pulmonary aspergillosis (IPA) when host defenses fail (Bulpa et al., 2007). Historically, IPA was regarded as a complication almost exclusive to neutropenia, a paradigm entrenched in clinical teaching and reflected in diagnostic algorithms that prioritized prolonged neutropenia as the defining risk factor. But the patient population has changed. Non-neutropenic cases are now climbing steadily, year on year (Latgé and Chamilos, 2019). The death rate among non-neutropenic IPA patients can match or even surpass that seen in classic high-risk groups (Cornillet et al., 2006; Bassetti et al., 2018). That alone makes early detection and treatment essential. The literature is clear on one point: neutropenia worsens prognosis in IPA (Gallien et al., 2008; Yan et al., 2009). Beyond that, for patients with normal counts, the factors that shape survival are poorly understood. That gap needs filling. IPA has been studied extensively, but mostly in the immunocompromised. Data on patients with intact immune systems and normal neutrophil counts are still thin (De Pauw et al., 2008; Kosmidis and Denning, 2015; Patterson et al., 2016; Ullmann et al., 2018). Most existing studies are focused on single-center retrospective analyses, and the risk factors involved (e.g., diabetes, respiratory diseases, corticosteroid exposure, etc.) vary considerably across different studies (Russo et al., 2011; Lugosi et al., 2014; He et al., 2024).

Bacterial co-infections may matter just as much as steroids, yet they have received far less attention. Gram-negative respiratory infections are common in ICU patients, and they can reshape the lung in ways that let Aspergillus take hold (Liu et al., 2025, 2026). The *P. aeruginosa*–*A. fumigatus* pairing has drawn growing interest. Both organisms share the airways of patients with chronic purulent lung disease—COPD, bronchiectasis, cystic fibrosis. In the test tube, *P. aeruginosa* can hold back A. fumigatus growth by pumping out phenazines and other diffusible molecules. Yet in the clinic, co-infection sometimes makes things worse, not better (Reece et al., 2018; Scott et al., 2019). Steroids usually raise IPA risk, but the story may change with the bacterium in the room. Jordana-Lluch et al. found that budesonide and fluticasone propionate reshape *P. aeruginosa*—thicker biofilm, toned-down virulence (Jordana-Lluch et al., 2025). Whether this bacterial reshaping alters fungal risk, and how, is still largely unknown.

Machine learning offers a pathway to integrate heterogeneous clinical, laboratory, and microbiological signals into an interpretable bedside tool. However, its application to IPA prediction in non-neutropenic populations has been limited, and few studies have combined algorithmic development with external validation and mechanistic interpretation (Liu et al., 2025). Realizing the translational potential of this approach calls for three things: strong discrimination, interpretability that clinicians can follow, and proof of clinical utility. Black-box models fall short on the second count; conventional risk scores fall short on the first. Closing this divide demands methods that do not sacrifice predictive accuracy for the sake of transparency, or vice versa.

This retrospective cohort had four objectives: to develop and externally validate a machine learning classifier for IPA in non-neutropenic patients; to apply SHAP analysis to identify key predictors, with specific attention to corticosteroid exposure; to test whether the corticosteroid-IPA association differs by bacterial co-pathogen, particularly *P. aeruginosa* versus other Gram-negative organisms; and to derive a simplified bedside score requiring no specialized software.

## Methods

### Predictor selection

We began with more than 60 candidate variables but chose not to enter all of them into the model, aiming to keep the classifier both interpretable and clinically useful. Through literature review and clinical judgment, we narrowed this pool to 20 predictors felt to be most pertinent to IPA. Core exposures—corticosteroids and *Pseudomonas aeruginosa*—were retained by default. We also kept established risk factors such as age, diabetes, chronic lung disease, and immune markers. Where variables overlapped in meaning or were tightly correlated, we dropped the less informative one: neutrophils gave way to lymphocytes, and CRP was preferred over other acute-phase reactants. Finally, we required that any retained variable be consistently recorded at all three centers with minimal missing data. This pre-selection process was performed independently of any outcome data, thus introducing no data-driven bias and adhering to the TRIPOD (Collins et al., 2024) statement recommendations on pre-selection of predictors.

### Study design and participants

From December 2020 through December 2025, we consecutively enrolled non-neutropenic patients hospitalized for pulmonary infections at three tertiary hospitals in China for this multicenter retrospective cohort study. Ethics clearance was obtained, and because the study was retrospective, the requirement for informed consent was waived.

Eligible participants were adults aged ≥18 years who met the IPA diagnostic criteria from established international guidelines (Bassetti et al., 2024). Confirmed IPA required histopathologic evidence of hyphal invasion from a sterile site or a positive culture of *Aspergillus spp.* from a sterile specimen. Probable IPA required fulfillment of all three domains: host factors, clinical and radiologic features, and mycologic evidence (positive Aspergillus culture from BALF, or galactomannan optical density index >0.5 in serum or ≥1.0 in BALF). Patients with profound immunosuppression, including solid-organ or hematopoietic stem-cell transplant recipients, were excluded.

### Machine learning model development

The discovery cohort (Centers A and B, n = 565) was split by stratified random sampling into a training set (70%, n = 396) and an internal validation set (30%, n = 169). Four classifiers were compared, with Random Forest selected for its superior discriminatory performance. Hyperparameters were tuned via 5-fold cross-validation, and inverse frequency weighting was applied to address class imbalance. Discrimination was measured by AUC, calibration by Brier score and calibration plots, and clinical utility by decision curve analysis. Optimism-corrected estimates were derived from 1000 bootstrap iterations.

The trained model was then applied unchanged to the external validation cohort (Center C, n = 272). Feature importance was assessed using SHAP values. The six highest-ranking predictors were transformed into a simple additive bedside score, with integer weights proportional to their mean absolute SHAP values: *P. aeruginosa* isolation (3 points), PaO_2_/FiO_2_ < 200 mmHg (2 points), diabetes mellitus (1 point), SOFA > 5 (1 point), lymphocyte count < 0.8 × 10^9^/L (1 point), and ICU admission (1 point). The high-risk cutoff (≥4 points) was determined by maximizing the Youden index; because every component weight is an integer, ≥3.5 is arithmetically identical to ≥4.

### Corticosteroid–bacteria interaction analysis

Patients were divided into three bacterial categories-*P. aeruginosa*, other Gram-negative bacilli, and non-Gram-negative organisms and the association between corticosteroids and IPA was examined separately within each category. Propensity scores were estimated for each subgroup, followed by 1:1 nearest-neighbor matching with a caliper of 0.2 SD, balancing age, sex, diabetes, chronic lung disease, APACHE II score, and SOFA score between corticosteroid-exposed and unexposed patients. The synergy index S was then calculated as a measure of biological interaction.

### Statistical analysis

All analyses were performed using R v4.5.3. Categorical variables are presented as n (%), continuous variables as mean ± SD or median (IQR) depending on distribution. A two-sided P < 0.05 was considered statistically significant.

### Data availability

The datasets developed and/or analyzed in this study are not publicly available due to ethical restrictions concerning patient privacy and anonymity. However, requests for access to the de-identified data can be submitted to the corresponding author (J.Y.Z.) under appropriate conditions and with institutional ethics committee approval.

## Results

### Baseline characteristics of the study population

A total of 837 patients were included, with 565 assigned to the discovery cohort and 272 to the external validation cohort (**Figure 1**). **Table 1** summarizes the baseline characteristics. Compared with the validation cohort, patients in the discovery cohort were older (median 70 vs. 65 years, P < 0.001), had a higher rate of ICU admission (44.2% vs. 29.4%, P < 0.001), and reported smoking more often (47.3% vs. 25.4%, P < 0.001). They also received corticosteroids (55.2% vs. 40.8%, P < 0.001) and antibiotics (60.9% vs. 48.9%, P = 0.001) more frequently. Higher levels of lymphocytes, interleukin-6, BALF galactomannan, and BALF 1,3-β-D-glucan were observed in the discovery cohort, along with more frequent fever, dyspnea, hemoptysis, nodular shadows, and bilateral lung involvement (all P < 0.05). The proportion of IPA was 43.0% in the discovery cohort versus 36.4% in the validation cohort (P = 0.068). Diabetes and chronic respiratory disease were more prevalent in the discovery cohort (both P < 0.001), whereas CURB-65 scores were higher in the validation cohort (P = 0.009). In-hospital mortality reached 19.6% in the discovery cohort and 12.1% in the validation cohort (P = 0.007).

**Figure 1:**
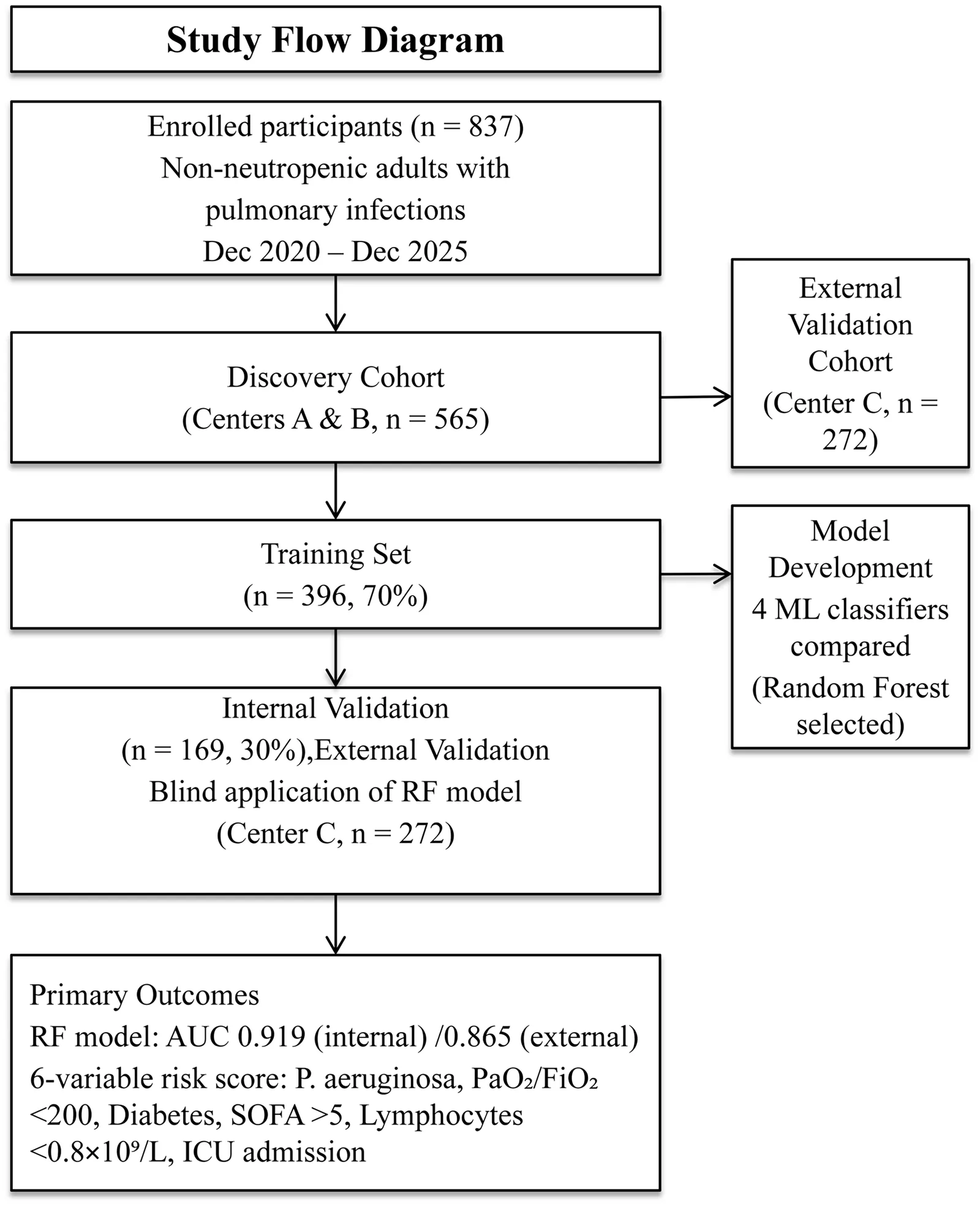
Study design and participant flow in the multicenter non-neutropenic cohort.

**Table 1:** Demographic and clinical characteristics of patients in the discovery cohort and external validation cohorts.

Variable	Discovery cohort (*n*=565)	External validation cohort (*n*=272)	χ^2^/Z	*P*
Age, years	70.00 (61.00, 76.00)	65.00 (54.75, 75.00)	3.782	<0.001
Male	338 (59.8%)	176 (64.7%)	1.847	0.174
Body mass index (kg/m²)	24.03 (22.03, 25.39)	24.09 (22.04, 25.77)	-0.658	0.511
PaO_2_/FiO_2_ ratio	234.00 (195.00, 272.00)	230.00(186.00,269.2)	1.613	0.107
ICU admission	250 (44.2%)	80 (29.4%)	16.922	<0.001
Smoking history	267 (47.3%)	69 (25.4%)	36.611	<0.001
Corticosteroid exposure	312 (55.2%)	111 (40.8%)	15.257	<0.001
Hormone.dosage	270.00 (0.00, 525.00)	0.00 (0.00, 471.25)	2.519	0.007
Corticosteroid.exposure.time	7.00 (0.00, 16.00)	0.00 (0.00, 15.00)	3.732	<0.001
Organ failure	229 (40.5%)	109 (40.1%)	0.016	0.899
Antibiotic treatment	344 (60.9%)	133 (48.9%)	10.765	0.001
Laboratory examination				
White blood cells (×10^9^)	8.33 (5.77, 12.18)	8.84 (5.66, 12.28)	-0.404	0.686
Neutrophils (×10^9^)	6.54 (4.19, 9.67)	7.53 (4.10, 11.77)	-2.149	0.032
Lymphocytes (×10^9^)	1.65 (1.13, 2.76)	0.90 (0.44, 1.55)	9.637	<0.001
Eosinophil(%)	0.02 (0.00, 0.09)	0.01 (0.00, 0.12)	0.656	0.499
Procalcitonin (ng/mL)	0.58 (0.11, 3.00)	0.56 (0.16, 2.21)	-0.536	0.592
Interleukin-6 (pg/mL)	9.00 (2.84, 26.00)	12.85 (4.26, 41.05)	-3.825	<0.001
C-reactive protein(mg/L)	15.90 (5.00, 44.40)	16.65 (7.16, 48.25)	-1.379	0.168
Erythrocyte sedimentation rate(mm/h)	12.00 (5.00, 29.00)	14.00 (6.00, 28.00)	-0.427	0.669
Hemoglobin(g/L)	105.00 (90.00, 130.00)	107.50(88.75, 130.25)	-0.05	0.959
GM assay (BALF)	1.22 (0.39, 2.89)	0.52 (0.36, 1.49)	4.394	<0.001
1,3-β-D-glucan assay (BALF)	41.00 (34.00, 59.79)	37.00 (33.00, 42.25)	6.702	<0.001
Lactic acid(mmol/L)	1.60 (1.00, 2.90)	1.60 (1.10, 3.02)	-1.00	0.317
Platelet count (×10^9^)	151.00 (90.00, 218.00)	153.00(89.50, 214.25)	0.031	0.976
Symptoms				
Fever	292 (51.7%)	111 (40.8%)	8.694	0.003
Shortness of breath	369 (65.3%)	121 (44.5%)	32.807	<0.001
Chest pain	82 (14.5%)	60 (22.1%)	7.421	0.006
Cough	433 (76.6%)	196 (72.1%)	2.061	0.151
Chest tightness	323 (57.2%)	154 (56.6%)	0.023	0.880
Hemoptysis	73 (12.9%)	9 (3.3%)	19.194	<0.001
Dyspnea	232 (41.1%)	86 (31.6%)	6.952	0.008
Chest imaging				
Pleural effusion	258 (45.7%)	130 (47.8%)	0.335	0.563
Nodular shadow	187 (33.1%)	67 (24.7%)	6.072	0.014
Patchy shadow	312 (55.2%)	134 (49.3%)	2.617	0.106
Cavity	92 (16.3%)	31 (11.4%)	3.497	0.062
Lung consolidation	337 (59.6%)	151 (55.5%)	1.289	0.256
Bilateral pulmonary involvement	154 (27.3%)	54 (19.9%)	5.389	0.020
Pleural thickening	346 (61.2%)	147 (54.0%)	3.926	0.048
Disease				
IPA	243 (43.0%)	99 (36.4%)	3.322	0.068
Gram negative infection	99 (17.5%)	61 (22.4%)	2.856	0.091
*Pseudomonas aeruginosa*	190 (33.6%)	94 (34.6%)	0.071	0.790
Gram positive infection	276 (48.8%)	117 (43.0%)	2.51	0.113
Hypoproteinemia	278 (49.2%)	119 (43.8%)	2.19	0.139
Diabetes	268 (47.4%)	84 (30.9%)	20.641	<0.001
Hypertension	264 (46.7%)	128 (47.1%)	0.008	0.928
Coronary heart disease	134 (23.7%)	62 (22.8%)	0.087	0.768
Respiratory disease	271 (48.0%)	89 (32.7%)	17.407	<0.001
Chronic liver disease	149 (26.4%)	55 (20.2%)	3.769	0.052
Kidney injury	156 (27.6%)	66 (24.3%)	1.055	0.304
Septicshock	111 (19.6%)	47 (17.3%)	0.672	0.413
Outcome				
In-hospital mortality	111 (19.6%)	33 (12.1%)	7.277	0.007
Hospital.LOS	12.00 (8.00, 18.00)	13.00 (8.00, 18.00)	-0.141	0.888
Invasive Mechanical Ventilation	158 (28.0%)	72 (26.5%)	0.206	0.650
Clinical severity scores				
SOFA score	9.00 (8.00, 11.00)	9.00 (8.00, 10.00)	1.907	0.053
CURB-65 score	2.00 (2.00, 3.00)	3.00 (2.00, 3.00)	-2.485	0.009
APACHE II score	12.00 (10.00, 14.00)	12.00 (10.00, 14.00)	-0.354	0.722

Data are presented as mean±SD, median (IQR), or n (%). *P* values were calculated using the Mann-Whitney U test for continuous variables and the χ² test or Fisher's exact test for categorical variables.

APACHE II, Acute Physiologic Assessment and Chronic Health Evaluation II; ICU, intensive care unit; PaO_2_/FiO_2_, arterial oxygen partial pressure/fractional inspired oxygen; LOS, length of stay; CURB-65, Confusion, Urea, Respiratory rate, Blood pressure, Age ≥65 years; SOFA, Sequential Organ Failure Assessment; BALF, bronchoalveolar lavage fluid.

### Model development and internal assessment

We divided the discovery cohort into training (70%) and internal validation (30%) sets by stratified random sampling. Of the four algorithms tested, Random Forest showed the best discrimination in the training set (AUC 0.919, F1 0.832, sensitivity 0.931, specificity 0.762; **Supplementary Table S1A**). Its calibration and cross-validated performance are summarized in **Supplementary Table S1B**: the 10-fold cross-validated training AUC was 0.915, with a Brier score of 0.127; in the internal validation set the AUC was 0.859, Brier score 0.152, and calibration slope 1.11; in the external validation cohort the AUC was 0.865, Brier score 0.143, and calibration slope 1.12. The higher training calibration slope (1.87) reflects shrinkage of cross-validated ensemble predictions rather than miscalibration. The ROC curve (**Figure 2A**), decision curve (**Figure 2B**), and calibration plot (**Figure 2C**) together pointed to good discrimination, clinical utility, and calibration with no clear pattern of over- or under-estimation.

**Figure 2:**
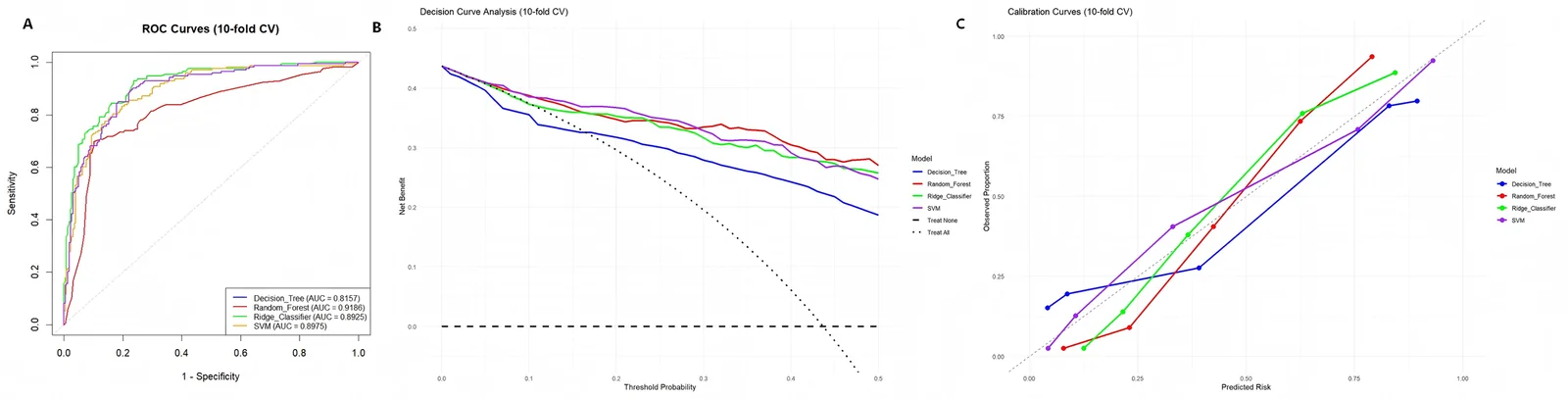
Performance of four machine learning classifiers for IPA prediction in the training cohort. **(A)** Receiver operating characteristic curves with AUC values. **(B)** Decision curve analysis showing net benefit across threshold probabilities. **(C)** Calibration plots of predicted versus observed IPA probabilities.

### External validation of the Random Forest model

We applied the Random Forest model fitted on Centers A and B to Center C. Discrimination held up well, with an AUC of 0.865 (**Figure 3A**). Decision curve analysis showed net clinical benefit across the range of threshold probabilities that matter in practice (**Figure 3B**). Predicted and observed risks agreed reasonably well, indicating acceptable calibration (**Figure 3C**).

**Figure 3:**
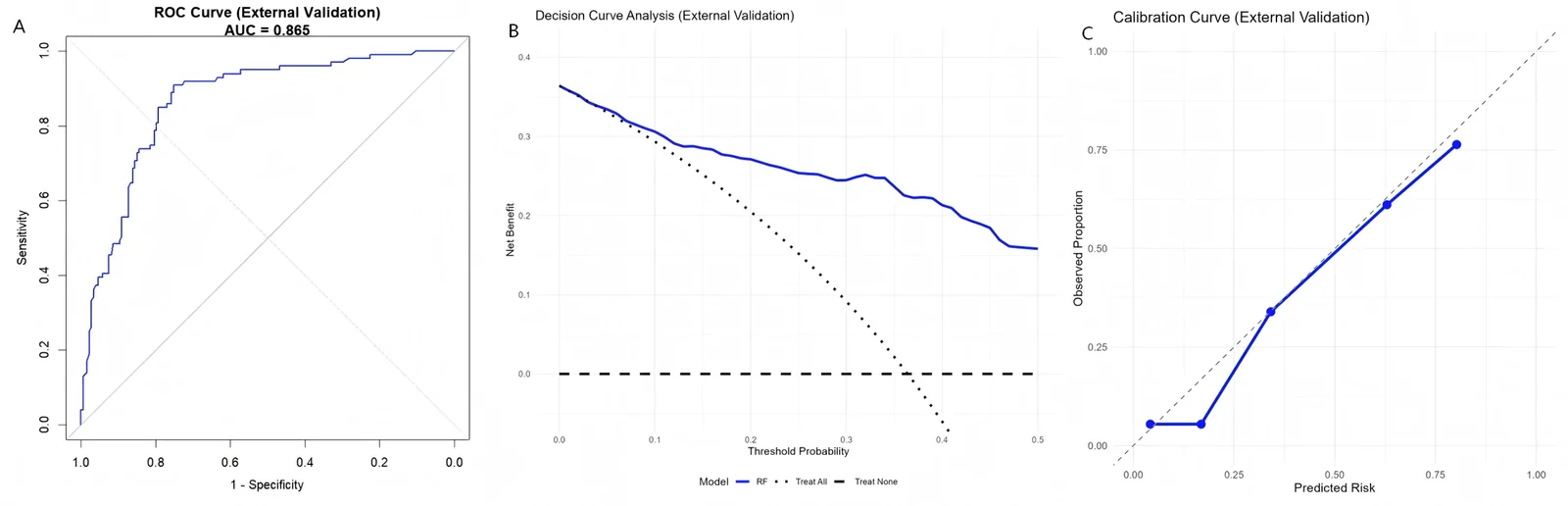
External validation of the optimal machine learning model in an independent cohort. **(A)** ROC curve and AUC in the external validation set. **(B)** Decision curve analysis for clinical net benefit. **(C)** Calibration curve assessing agreement between predicted and observed risks.

### Feature importance and derivation of a simplified risk score

SHAP analysis left six variables standing (**Figure 4**). *Pseudomonas aeruginosa* dominated; the remaining five—PaO_2_/FiO_2_ under 200, diabetes, SOFA over 5, lymphocytes below 0.8 × 10^9^/L, and ICU admission—trailed well behind. These six predictors were converted into a six-variable additive score with integer weights proportional to their mean absolute SHAP values (**Supplementary Table S2A**): *P. aeruginosa* isolation (3 points), PaO_2_/FiO_2_ <200 mmHg (2 points), diabetes mellitus (1 point), SOFA >5 (1 point), lymphocyte count <0.8 × 10^9^/L (1 point), and ICU admission (1 point). The score ranges from 0 to 9, and the high-risk cutoff was set at ≥4 points. The missing corticosteroid was striking: steroids are widely feared in this context, but the algorithm did not rank them among the top predictors.

**Figure 4:**
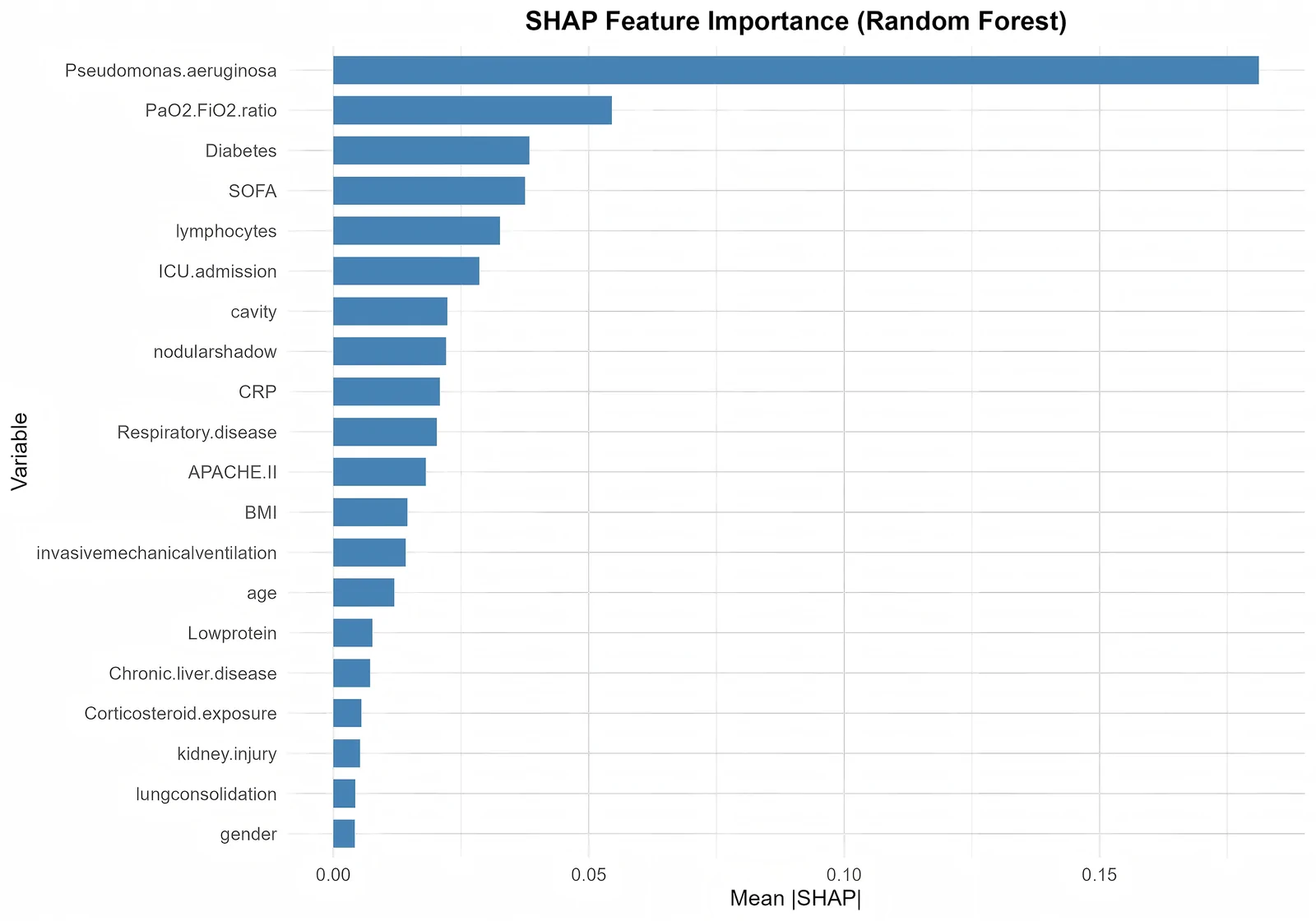
Feature importance for IPA prediction derived from SHAP values.

### Performance of the simplified risk score across cohorts

The six-variable score was evaluated in all three cohorts (**Supplementary Table S2B**; **Supplementary Figures S1A–C**). At the ≥4 cutoff, AUCs were 0.869 in training, 0.774 in internal validation, and 0.719 in external validation; corresponding sensitivity/specificity were 0.832/0.803, 0.714/0.677, and 0.768/0.624. Full operating characteristics, including PPV, NPV and Youden index, are reported in **Supplementary Table S2B**. Because the cutoff was derived in the internal validation set, those estimates are slightly optimistic, whereas the external-validation estimates are least biased. Decision curve analysis showed net benefit across 5–35% risk thresholds, and calibration plots showed no marked over- or underestimation (**Supplementary Figures S1B, C**).

### Corticosteroid–bacteria interaction and subgroup analyses

The association between corticosteroid use and IPA was examined separately for each bacterial category (**Supplementary Table S3**). In adjusted analyses, no subgroup showed a statistically significant effect: for *P. aeruginosa*, OR 0.524 (95% CI 0.214–1.283, P = 0.157); for other Gram-negative bacteria, OR 2.172 (95% CI 0.722–6.535, P = 0.168); and for non-GNB, OR 0.803 (95% CI 0.443–1.454, P = 0.468). The overall OR was 1.208 (95% CI 0.833–1.752, P = 0.318). The synergy index S was 0.041. The formal test for effect modification was not statistically significant (P for interaction = 0.131, likelihood-ratio test, 2 df).

The direction of the corticosteroid effect varied across bacterial pathogens (**Supplementary Figure S2**). In the unadjusted analysis (**Supplementary Figure S2A**), *P. aeruginosa* showed an OR below 1 (OR 0.52), other Gram-negative bacteria an OR above 1 (OR 2.17), and non-GNB a neutral estimate (OR 0.80). After propensity score matching (**Supplementary Figure S2B**; matched for age, sex, diabetes, chronic lung disease, APACHE II, and SOFA), this directional pattern persisted: OR for *P. aeruginosa* 0.60 (95% CI 0.22–1.65), for other Gram-negative bacteria 1.75 (95% CI 0.51–5.98), and for Gram-positive bacteria 0.91 (95% CI 0.50–1.67). Although confidence intervals widened and none reached statistical significance, the divergent directions—OR below 1 with *P. aeruginosa* versus OR above 1 with other Gram-negative bacilli-remained consistent.

### Twenty-eight-day survival stratified by bacterial subgroup

Kaplan-Meier curves are shown in **Supplementary Figure S3**. In the discovery cohort, patients with non-pseudomonal GNB had the highest mortality. Their survival probability separated from the *P. aeruginosa* and non-GNB groups by days 7-10 (log-rank P < 0.001). The external validation cohort reproduced this pattern: the non-pseudomonal GNB subgroup again showed the lowest survival, while the *P. aeruginosa* and non-GNB curves overlapped in both cohorts. These comparable outcomes suggest no survival penalty associated with *P. aeruginosa* isolation.

## Discussion

Unlike patients with neutropenia, those without it frequently show subtle or indolent clinical features, which often lead to underrecognition, delayed diagnosis, and an increased mortality linked to the disease (Cornillet et al., 2006).

In this multicenter retrospective cohort study of 837 non-neutropenic patients hospitalized with pulmonary infections, we made several important observations regarding the intricate relationship between bacterial co-pathogens, corticosteroid exposure, and IPA. First, we developed and externally validated a Random Forest model that achieved good discriminatory performance for IPA prediction. Second, through SHAP-based feature importance analysis, corticosteroid exposure did not emerge among the top six predictors of IPA-a finding that contrasts with the well-established paradigm positioning corticosteroids as a major risk factor for aspergillosis (Donnelly et al., 2020). Third, the direction of the corticosteroid effect on IPA risk varied markedly with the accompanying bacterial pathogen: an OR below 1 was seen with *Pseudomonas aeruginosa* infection, whereas an OR above 1 emerged with other Gram-negative bacilli. Fourth, patients carrying non-pseudomonal Gram-negative infections had the poorest 28-day survival, while those with *P. aeruginosa* co-infection showed survival outcomes similar to patients without any Gram-negative infection. Taken together, these findings argue that the impact of corticosteroids on IPA risk is not consistent across different bacterial backgrounds. Instead, it may be critically shaped by which bacterial pathogen colonizes or infects the respiratory tract.

A 2023 systematic review and meta-analysis found that corticosteroid therapy was associated with worse clinical outcomes in invasive aspergillosis (HR: 2.50, 95%CI: 1.89–3.31, p < 0.001), with high-dose corticosteroids (more than 2 mg/kg/day of prednisolone equivalents) significantly increasing mortality (HR: 2.94, 95%CI: 2.13–4.05, p < 0.001) (Li and Denning, 2023). Mechanistically, glucocorticoids increase susceptibility to Aspergillus primarily by suppressing NF-kB-mediated neutrophil control of fungal hyphae. Thrikawala and colleagues demonstrated in a zebrafish model that dexamethasone acts through the glucocorticoid receptor to suppress NF-kB activation at the infection site, thereby impairing neutrophil restriction of invasive hyphal growth (Thrikawala et al., 2024).

Given this well-established body of evidence, the observation that corticosteroid exposure did not rank among the top six SHAP-derived predictors in our model warrants careful consideration. Several explanations may account for this apparent discrepancy. First, our population explicitly excluded profoundly immunosuppressed patients (e.g., solid-organ or hematopoietic stem-cell transplant recipients). In non-neutropenic ICU patients, other factors—such as chronic lung disease, diabetes, organ failure, and ICU admission—may assume relatively greater importance (Blot et al., 2012; Koulenti et al., 2014). Second, the preselection of variables based on literature review and clinical plausibility (which followed TRIPOD recommendations (Collins et al., 2024)) retained corticosteroid exposure as a core variable, but its absence from the SHAP top six may reflect the non-linear, dose-dependent nature of the relationship. Recent work by Liu and colleagues demonstrated that IPA risk peaks after 5–15 days of corticosteroid use, particularly when the cumulative dose exceeds 600 mg of prednisone equivalent (Liu et al., 2025). Our binary exposure variable (any versus no corticosteroid) could have obscured such non-linear threshold effects. Third, SHAP values measure what a predictor adds at the margin, given the particular model and data. When other strong clinical signals-ICU admission, low lymphocyte count, organ failure-are already in the mix, the steroid effect may partly dissolve into them, especially if steroids operate through these variables rather than independently.

The corticosteroid story turned out to be more complicated than a simple risk label. In patients carrying *P. aeruginosa*, steroids looked OR below 1; in those with other Gram-negative bacilli, the same exposure pointed toward OR above 1. We saw this pattern in the raw data, and it persisted after propensity matching. It also replicated in the external cohort, suggesting the split is not a training-set artifact. The explanation is unclear, but we suspect steroids may act directly on *P. aeruginosa*-dampening virulence programs that normally clear the path for Aspergillus. Pyocyanin output, quorum-sensing signaling, or type III secretion could all be affected. Budesonide and fluticasone propionate both cut IL-8 release by 15-50% and lowered bacterial invasiveness into host cells by 25-40%. Yet the same drugs also nudged the organism toward biofilm growth, with a 1.6-fold increase at peak concentration after sufficient exposure time (Jordana-Lluch et al., 2025).

At first glance, the OR below 1 linked to corticosteroids in *P. aeruginosa* carriers seems counterintuitive, especially given the widely held view that corticosteroids uniformly raise IPA risk. A deeper look at how *P. aeruginosa* and A. fumigatus naturally interact, however, offers a plausible biological explanation. These two pathogens are not passive co-occupants of the airways; instead, they engage in a complex, two-way antagonism that is now being unraveled at the molecular level.

Multiple lines of evidence indicate that *P. aeruginosa* possesses an intrinsic capacity to inhibit A. fumigatus through several independent mechanisms. First, *P. aeruginosa* secretes redox-active phenazines, including pyocyanin, 1-hydroxyphenazine, phenazine-1-carboxamide and phenazine-1-carboxylic acid, which suppress A. fumigatus growth by inducing oxidative stress and damaging mitochondrial ultrastructure. Sass et al. demonstrated that under nonlimiting iron conditions, pyocyanin becomes the major antifungal molecule, whereas under iron-limited conditions *P. aeruginosa* switches to an iron-denial strategy mediated by the siderophore pyoverdine (Sass et al., 2021). Second, *P. aeruginosa* produces the filamentous bacteriophage Pf4, which avidly binds to A. fumigatus hyphae and sequesters environmental iron, thereby depriving the fungus of this essential nutrient. This inhibition is dose-dependent and can be reversed by supplemental ferric iron, confirming that iron chelation is the operative mechanism (Penner et al., 2016). Third, the *P. aeruginosa* siderophore pyoverdine itself has been identified as a key inhibitory molecule against A. fumigatus biofilms, acting primarily through iron chelation. Sass et al. studied 24 *P. aeruginosa* mutants and concluded that pyoverdine is the central factor in this antifungal activity, with pure pyoverdine recapitulating the inhibitory effect (Sass et al., 2018). Fourth, Ferreira et al. showed that clinical isolates of *P. aeruginosa* inhibit the growth and biofilm formation of A. fumigatus from conidia, an effect that does not require direct bacterial contact and is mediated by secreted products in culture filtrates. Notably, isolates from cystic fibrosis patients were more inhibitory than non-CF isolates, and non-mucoid CF isolates showed the strongest activity (Ferreira et al., 2015).

In turn, A. fumigatus can reciprocally inhibit *P. aeruginosa* through the secretion of gliotoxin, a potent secondary metabolite that exerts strong anti-Pseudomonal and anti-biofilm activity, as reported by Reece et al. and others (Reece et al., 2018). Other studies have further shown that dirhamnolipids secreted by *P. aeruginosa* can block β1,3 glucan synthase activity in A. fumigatus, altering cell wall architecture and inhibiting fungal growth (Briard et al., 2017), and that exposure to *P. aeruginosa* secretomes induces profound proteomic changes in A. fumigatus, including altered gliotoxin production (Margalit et al., 2022). Thus, the overall IPA risk likely hinges on a balance between steroids’ direct suppression of host immunity and the indirect easing of fungal inhibition driven by *P. aeruginosa*. For patients carrying other Gram-negative bacilli that lack such a potent anti-Aspergillus repertoire, this anti-Aspergillus mechanism is absent, which may account for the OR above 1 steroid effect seen in those subgroups. Although this interpretation is still speculative and awaits direct experimental validation, it offers a coherent framework that bridges our clinical observation with a large body of mechanistic evidence on interspecies antagonism in the airway.

Survival analysis backed up the divergence we saw in risk. The non-pseudomonal Gram-negative group had the steepest decline; by days 7–10 their survival probability had already peeled away from the other two groups. This is consistent with the aggressive nature and mounting resistance of many Gram-negative pathogens in the ICU (Vincent et al., 2020). *P. aeruginosa* told a different story: survival in this group overlapped with the non-Gram-negative group, implying that *P. aeruginosa* isolation—at least in this cohort—did not translate into extra short-term mortality. The explanation likely lies in what *P. aeruginosa* actually is in these patients. It is typically a chronic colonizer of damaged airways—COPD and bronchiectasis being the classic backgrounds—not an acute pathogen storming a previously healthy lung. Against this backdrop, isolating *P. aeruginosa* says more about years of structural disease and illness severity than about a new lethal insult. Add to this the steroid effect: corticosteroids appear to dial down *P. aeruginosa* virulence in this setting (Jordana-Lluch et al., 2025), so the bacterium may not deliver the acute punch one would otherwise expect, which aligns with the comparable survival we observed.

Our findings have two clinical implications. First, IPA risk scores should incorporate bacterial data. Our six-variable score (*P. aeruginosa*, PaO_2_/FiO_2_ <200 mmHg, diabetes, SOFA >5, lymphocytes <0.8 × 10^9^/L, ICU admission) is suitable for bedside use, though its AUC (0.719) is lower than that of the full model. In a like-for-like comparison in the external cohort, the simplified score lost only 0.034 in AUC relative to a full multivariable logistic model containing the same 20 predictors (0.719 vs. 0.753; paired DeLong P = 0.181), indicating that most of the gap from the random forest reflects non-linearity rather than the reduction in predictor number. At a 20% threshold, the score retained 82% of the random forest’s net benefit. Second, steroid stewardship should account for bacterial context: in patients with *P. aeruginosa* co-infection, the assumption that corticosteroids uniformly increase IPA risk may not hold. The synergy index (S = 0.041) indicates that a steroid–*P. aeruginosa* interaction promoting IPA is unlikely. The divergent effects—OR below 1 with *P. aeruginosa* but OR above 1 with other Gram-negative bacilli—may reflect pathogen-specific biofilm remodeling or host immune responses, although these mechanisms remain speculative. The 2025 German ICU guideline for IPA lays out host factors, clinical features, and mycologic evidence as the diagnostic pillars (Wichmann et al., 2025). Bacterial co-pathogens are nowhere in that framework. That omission looks increasingly hard to defend, and our results give grounds for adding it. Working out which bacterial species turns the steroid effect toward OR below 1 versus OR above 1 would be a first step toward microbiome-informed prevention.

Our study has several limitations. Both cohorts were from Chinese tertiary hospitals, limiting generalizability to other populations. The retrospective design may have residual confounding despite matching. Steroid dose, timing, and cumulative exposure were unavailable, precluding assessment of non-linear effects. Respiratory cultures cannot reliably distinguish colonization from active infection. Subgroup analyses had small IPA events, yielding wide confidence intervals; results are hypothesis-generating. Exclusion of neutropenic patients limits the generalizability of our findings to neutropenic populations. Preselection of 20 predictors, though TRIPOD-adherent, may have missed informative variables. In sensitivity analyses, adjusting for chest-CT structural markers moved the OR toward 1 (0.63, 95% CI 0.24–1.61), and excluding patients with chronic respiratory disease gave an OR of 1.24 (95% CI 0.25–6.06, P for interaction = 0.355), indicating that the estimate is unstable and may reflect confounding by structural lung disease. The E-value for the confidence limit nearest the null was 1.89, indicating that unmeasured confounding alone could explain the estimate away.

Future work should prospectively validate the score in diverse ICU settings, elucidate corticosteroid–bacteria interactions via mechanistic models, and assess real-world impact through EHR integration and implementation research.

## Conclusions

We validated an interpretable machine learning model for IPA risk stratification in non-neutropenic patients. The corticosteroid–IPA association varied by bacterial co-pathogen: *P. aeruginosa* isolation was associated with an OR below 1, whereas other Gram-negative bacilli were associated with an OR above 1. These findings support pathogen-specific bedside tools over undifferentiated steroid-based risk labels, and suggest that future guidelines incorporate bacterial context.

## Data Availability

The datasets analyzed in this study are not publicly available due to ethical restrictions concerning patient privacy and anonymity. Requests for access to the deidentified data may be submitted to the corresponding author (J.Y.Z.) under appropriate conditions and with institutional ethics committee approval.
