# Supplementary material for: Machine learning-based risk prediction for invasive pulmonary aspergillosis in non-neutropenic intensive care unit patients, with an exploratory analysis of pathogen-related risk factors
Source: Front Cell Infect Microbiol. 2026 Oct 14;16:1967577. doi: 10.3389/fcimb.2026.1967577 (PMC13713394; doi:10.3389/fcimb.2026.1967577)
Supplement: Supplementary file 1 [file SupplementaryFile1.docx]

Supplementary Table S1a Classifier performance in the training set. AUC, F1 score, sensitivity, and specificity for the four candidate models

| Model | AUC | F1 | Sensitivity | Specificity |
| --- | --- | --- | --- | --- |
| Decision Tree | 0.816 | 0.763 | 0.699 | 0.897 |
| Random Forest | 0.919 | 0.832 | 0.931 | 0.762 |
| Ridge Classifier | 0.893 | 0.797 | 0.804 | 0.834 |
| SVM | 0.898 | 0.819 | 0.902 | 0.767 |

AUC 0.919 is the apparent/resubstitution estimate; the 10-fold cross-validated estimate is 0.915 (S1b).

Supplementary Table S1b. Discrimination and calibration of the Random Forest model.Model performance in the training, internal validation, and external validation cohorts.

| Cohort | n | AUC (95% CI) | Brier score | Calibration slope | Calibration intercept |
| --- | --- | --- | --- | --- | --- |
| Training (10-fold cross-validated predictions) | 396 | 0.915 | 0.127 | 1.87 | 0.154 |
| Internal validation | 169 | 0.859 | 0.152 | 1.11 | −0.168 |
| External validation | 272 | 0.865 | 0.143 | 1.12 | −0.205 |

The Brier score and calibration slope were computed on the predicted probabilities of the final Random Forest model (ntree = 500). For the training cohort, predictions are 10-fold cross-validated out-of-fold predictions rather than resubstitution predictions, which are optimistically biased; this is the same basis on which the training AUC was originally reported. The elevated training calibration slope (1.87) reflects the shrinkage inherent in cross-validated ensemble predictions and should not be read as miscalibration. In both independent cohorts the slope is close to 1, indicating satisfactory calibration. Calibration slope and intercept were estimated by logistic regression of the observed outcome on the logit of the predicted probability.

Supplementary Table S2a Feature importance for each of six parameters for our trained RF model

| Feature | Scoring | Scoring Rules |
| --- | --- | --- |
| Pseudomonas aeruginosa | 3 | positive |
| PaO_2_/FiO_2_ ratio | 2 | ＜200 |
| Diabetes | 1 | positive |
| SOFA | 1 | ＞5 |
| lymphocytes | 1 | ＜0.8 |
| ICU admission | 1 | positive |

Weights are integers proportional to the mean absolute SHAP values of each predictor. The score therefore ranges from 0 to 9 (3 + 2 + 1 + 1 + 1 + 1 = 9); the observed range in our cohort was 0–9. Because every weight is an integer, the previously stated cutoff of ≥3.5 points is arithmetically identical to ≥4 points. The high-risk cutoff of ≥4 was derived by maximizing the Youden index in the internal validation set and applied unchanged to the other cohorts (see Supplementary Table S2b).

Supplementary Table S2b. Operating characteristics of the six-variable score at the ≥4 cutoff.

| Cohort | n | IPA events | AUC | Sensitivity | Specificity | PPV | NPV | Youden index |
| --- | --- | --- | --- | --- | --- | --- | --- | --- |
| Training | 396 | 173 | 0.869 | 0.832 | 0.803 | 0.766 | 0.861 | 0.635 |
| Internal validation | 169 | 70 | 0.774 | 0.714 | 0.677 | 0.610 | 0.770 | 0.391 |
| External validation | 272 | 99 | 0.719 | 0.768 | 0.624 | 0.539 | 0.824 | 0.392 |

The AUC of the continuous score does not depend on the chosen cutoff. PPV, positive predictive value; NPV, negative predictive value. The cutoff was fixed at ≥4 after being derived in the internal validation set, so the internal validation operating characteristics are slightly optimistic; the external validation column is the unbiased estimate.

Supplementary Table S3. Association between corticosteroid exposure and IPA, stratified by bacterial category.

| Bacteria group | n | IPA events | OR | 95% CI | P value | P for interaction |
| --- | --- | --- | --- | --- | --- | --- |
| Pseudomonas aeruginosa | 190 | 157 | 0.524 | 0.214–1.283 | 0.157 | 0.131 |
| Other Gram-negative bacilli | 99 | 19 | 2.172 | 0.722–6.536 | 0.168 | 0.131 |
| Non-Gram-negative | 276 | 67 | 0.803 | 0.443–1.454 | 0.468 | 0.131 |
| Overall | 565 | 243 | 1.208 | 0.833–1.752 | 0.318 | — |

Odds ratios are adjusted for age, sex, diabetes mellitus, chronic respiratory disease, APACHE II score, and SOFA score. The "P for interaction" was obtained by likelihood-ratio test (2 degrees of freedom) comparing a model containing a corticosteroid × bacterial-category interaction term with the corresponding main-effects model. The test was not statistically significant (P = 0.131), and no individual subgroup reached statistical significance; the subgroup estimates are therefore reported as exploratory and hypothesis-generating. Because the confidence interval for *P. aeruginosa* already includes 1, the estimate can be explained by unmeasured confounding without invoking any biological effect; the corresponding E-value for the confidence limit nearest the null is 1.89. The Overall row is derived from a model fitted without the bacterial-category term, so that it represents the sample-wide corticosteroid association rather than a category effect. Firth penalisation was not required; the model converged without separation.
