# Supplementary material for: Machine learning-based risk prediction for invasive pulmonary aspergillosis in non-neutropenic intensive care unit patients, with an exploratory analysis of pathogen-related risk factors
Source: Front Cell Infect Microbiol. 2026 Oct 14;16:1967577. doi: 10.3389/fcimb.2026.1967577 (PMC13713394; doi:10.3389/fcimb.2026.1967577)
Supplement: Supplementary file 2 [file SupplementaryFile2.docx]

**Supplementary Figure Legends**

Supplementary Figure S1. Discrimination of the simplified risk scoring system across three datasets. (A) ROC curves of the risk score in training, internal validation, and external validation sets (with AUCs). (B) Decision curve analysis for the risk score across the three cohorts. (C) Calibration curves comparing predicted versus observed IPA events.

Supplementary Figure S2. Subgroup‑specific effects of corticosteroid exposure on IPA stratified by bacterial type. (A) Forest plot of corticosteroid effect on IPA. (B) PSM-based effect of corticosteroid exposure on IPA within each bacterial group.

Supplementary Figure S3. Twenty-eight-day survival by bacterial subgroup. (A) Training and internal validation cohorts. (B) External validation cohort.
